# Multiple slips impact in the MHD hybrid nanofluid flow with Cattaneo–Christov heat flux and autocatalytic chemical reaction

**DOI:** 10.1038/s41598-021-94187-4

**Published:** 2021-07-16

**Authors:** Hina Gul, Muhammad Ramzan, Jae Dong Chung, Yu-Ming Chu, Seifedine Kadry

**Affiliations:** 1 Department of Computer Science, Bahria University, Islamabad, 44000 Pakistan; 2 Department of Mechanical Engineering, Sejong University, Seoul, 143-747 Korea; 3 Department of Mathematics, Huzhou University, Huzhou, 313000 People’s Republic of China; 4 Hunan Provincial Key Laboratory of Mathematical Modeling and Analysis in Engineering, Changsha University of Science & Technology, Changsha, 410114 People’s Republic of China; 5 Faculty of Applied Computing and Technology, Noroff University College, Kristiansand, Norway

**Keywords:** Software, Mechanical engineering

## Abstract

The present study deliberates the nanofluid flow containing multi and single-walled carbon nanotubes submerged into Ethylene glycol in a Darcy–Forchheimer permeable media over a stretching cylinder with multiple slips. The innovation of the envisaged mathematical model is enriched by considering the impacts of non-uniform source/sink and modified Fourier law in the energy equation and autocatalytic chemical reaction in the concentration equation. Entropy optimization analysis of the mathematical model is also performed in the present problem. Pertinent transformations procedure is implemented for the conversion of the non-linear system to the ordinary differential equations. The succor of the Shooting technique combined with the bvp4c MATLAB software is utilized for the solution of a highly nonlinear system of equations. The impacts of the leading parameters versus engaged fields are inspected through graphical sketches. The outcomes show that a strong magnetic field strengthens the temperature profile and decays the velocity profile. Also, the fluid velocity is lessened for growing estimates of the parameter of slip. Additionally, it is detected that entropy number augmented for higher thermal relaxation parameter and Reynolds number. To substantiate the existing mathematical model, a comparison table is also added. An excellent correlation is achieved here.

## Introduction

Carbon nanotubes (CNTs) were originally coined in 1991. CNTs with a diameter of 0.7–50 nm are thin cylinder-shaped made up of pure carbon. The role of CNTs is fundamental in many modern applications including nanotechnology, conductive plastics, composite materials, atomic force microscope, composite materials, and antifouling shades, electromagnetic gadgets, high-temperature refractors, medical device biosensors, and radio antennas. Two renowned types of CNTs i.e., single wall (SWCNTs) and multi-wall (MWCNTs) are recognized in the literature. Researchers have shown great interest in the studies focusing CNTs. Khan et al.^1^ found the numerical solution of three-dimensional nanofluid flow comprising CNTs with Cattaneo–Christov heat flux (C–C) in a Darcy–Forchheimer porous media with velocity and thermal slip conditions. The MATLAB function bvp4c technique is employed in this exploration. It is understood that large values of the respective slip parameter increase the axial velocity nevertheless a decline in the temperature field is observed. Furthermore, it is also observed that the Surface drag coefficient shows opposite behavior for porosity parameter and nanofluid volume fraction. Ramzan et al.^2^ explored numerical solution of entropy generation for carbon nanotubes with melting heat past over a swirling cylinder. It is revealed here that for expended estimations of Brinkman number and magnetic parameter, entropy is increased. Impact of MHD C–C heat flux with second-grade liquid past a starching cylinder studied by Alamri et al.^3^. It is noted that the velocity is weakened for large estimates of magnetic parameter and an opposing behavior is noted for the fluid parameter. The nanofluid flow containing CNTs with C–C heat flux in a stratified media over a rotating channel is discussed by Ramzan et al.^4^. The numerical solution of the proposed model is found by employing the MATLAB function using the bvp4c function. It is observed that the temperature profile is declined for mounting estimates of the stratification parameter. The influence of Surfactant Type and Nanoparticle Concentration on Thermal Performance with Bauxite nanoliquid discussed by Aydin et al.^5^. Sozen et al.^6–8^ examine the performance of nanoliquid in a heat exchanger plate numerically and experimentally. It is used Tio-deionized and deionized water nanoliquid for greater heat transfer rate. It is also studied the effects of fly ash and alumina nanoliquid employed on the performance of heat pipe bundle with recuperator. Zeeshan et al.^9^ explored Natural convection heat transfer nanofluid flow analysis with water and CNT under the vertical truncated wavy cone. It is noted that by enhancing the CNT volume fraction thermal boundary thickness is reduced. Zeeshan et al.^10^ studied the impacts of Ohmic dissipation, thermal radiation, magnetic field and electric field with convective Poiseuille nanofluid flow over a porous wavy channel. Some more examinations featuring CNT’s might be found at^11–21^.

Synthetization of hybrid nanofluid depend upon the shape, volume fraction, and temperature. The goal of constructing hybrid nanofluids is to boost thermal conductivity. The thermal conductivity has been found tremendous application and wide range of physical necessities for example extraction of crude oil, geothermal mechanism, tinning of metal wires, fuel cells, and nuclear reactors, etc. Maskeen et al.^22^ introduced the enhancement of heat transfer rate of hybrid nanofluid flow in copper-alumina with water as a base fluid past over a stretching cylinder. It is observed the different effects of thermal radiation by employing Roseland’s flux model. The hybrid nanoliquid flow and MHD with stagnation point past over a circular cylinder examine by Nadeem et al.^23–25^. It is observed that for hybrid nanoliquid rate of heat transfer is high as compared to nanoliquid. It also discussed the thermal and velocity slip effects. The influence of Cattaneo–Christov heat flux and HOM-HET reactions with hybrid nanoliquid flow past over a cylinder explored by Christopher^26^. It is noticed that the thermal profile decreases for higher thermal relaxation time while increases for greater porosity parameter. A significant number of researchers have concentrated on the visualization of thermophysical features of hybrid nanofluids^27–30^.

It is a very much perceived fact that the transfer of heat phenomenon happens due to temperature inconsistency between two different objects or within the same object. The Fourier law (conduction of heat)^31^ was considered as a thumb rule in heat transfer processes for almost one century. But a major drawback of this law was that an initial disruption carries out through the process which denies the causality principle. To resolve this issue, Cattaneo^32^ inserted thermal relaxation time term in the customary Fourier's law (conduction of heat) which strengthens the transport of heat by the propagation of thermal waves with restricted speed and it was arduous to get a single heat equation. Later, Christov^33^ upgraded the Cattaneo model by inserting the upper-convected Oldroyd’s derivative and got a single equation for the temperature profile. This revised model is labeled as the (C–C) heat flux model. Khan et al.^34^ examined the unsteady Maxwell liquid flow with C–C heat flux over a stretching cylinder. The effects of Carreau liquid flow in two-dimensional flow with generalized Fourier and Fick’s law explored by Khan et al.^35^. It observed that the temperature profile is declined for the Brownian motion, Prandtl number, and curvature parameter. Ramzan et al.^36^ introduced C–C impact on Tangent hyperbolic liquid flow with second-order slip. The Runge–Kutta Fehlberg method is utilized to solve the problem. The analytic solution of visco-elastic in the existence of C–C thermal flux and velocity-slip is explored by Han et al.^37^. Ramzan et al.^38^ scrutinized the third-grade fluid flow with homogeneous–heterogeneous reactions in the existence of C–C heat flux. Analytical results by utilizing the HAM are obtained. It is revealed that temperature profile is an increasing function for Biot number. Some more recent attempts discussing C–C heat flux may be found at references^39–42^.

Chemical reactions have numerous applications and are classified as homogeneous–heterogeneous (HOM and HET) reactions. The role of the catalyst becomes fundamental if the reaction rate is comparatively slow. To some extent, the association of the HOM and HET reactions is bewildering. As the reaction and consumption rate of the reactants fluctuate with time. The applications of chemical reactions may be found in a variety of processes including polymers, the formation of fog, and crop damage owing to the freezing atmosphere. Hayat et al.^43^ studied the MHD Powell-Eyring fluid flow with HOM–HET reactions and Newtonian heating past a stretching cylinder. It is found here that HOM–HET parameters have an inverse impact on concentration profile. The Williamson liquid flow with HOM–HET reactions over an extending cylinder is scrutinized by Malik et al.^44^. The problem is explained using the Keller box, an implicit finite difference technique. Hayat et al.^45^ talked about the flow of Jeffrey nanofluid with the effect of HOM and HET reactions and C–C heat flux. A computational model of 3D water-CNTs amalgamation nanofluid flow with HOM-HET reactions is studied by Hayat et al.^46^. An analytical solution is found by implementing the Homotopy analysis scheme. The results indicate that for higher nanoparticle volume fraction, the skin friction coefficient components are enhanced. Lu et al.^47^ debated the unsteady fluid flow containing single and multi-walled CNTs in the attendance of C–C heat flux and HOM–HET reactions amidst two rotating disks. The numerical solution is obtained by utilizing the bvp4c function of the MATLAB software. More studies highlighting the role of HOM–HET reactions are appended at^48–51^.

The aforementioned studies disclose that rare investigations are deliberated in the literature that signifies the nanofluid flow with immersed carbon nanotubes and HOM–HET reactions over varied geometries. However, no research so far carried out that interconnects the impact of multiple slips with modified Fourier law with non-uniform source/sink in a Darcy–Forchheimer spongy media over a stretching cylinder. The entropy optimization analysis is also a part of this study that boosts the uniqueness of the proposed model. The numerical results of the problem are found. To highlight the novelty of the presented model with the contemporary studies, Table 1 is formed. The uniqueness of the existing study is obvious.

**Table 1:** Comparison of the present model with the contemporary studies.

	Multiple slips	Hybrid nanofluid	Cattaneo–Christov heat flux	HOM–HET chemical reaction	Entropy analysis	Non-uniform, heat source/sink	Porous media	Darcy effect	CNT
Kumar et al.^52^	No	Yes	Yes	No	No	No	No	No	No
Christopher et al.^53^	No	Yes	Yes	Yes	No	No	No	No	No
Giri et al.^26^	No	No	No	Yes	No	No	No	Yes	Yes
Hussain et al.^54^	No	No	No	Yes	No	No	Yes	Yes	No
Lui et al.^55^	Yes	No	No	No	No	No	Yes	Yes	No
Present	Yes	Yes	Yes	Yes	Yes	Yes	Yes	Yes	Yes

### Mathematical formulation

**Figure 1:**
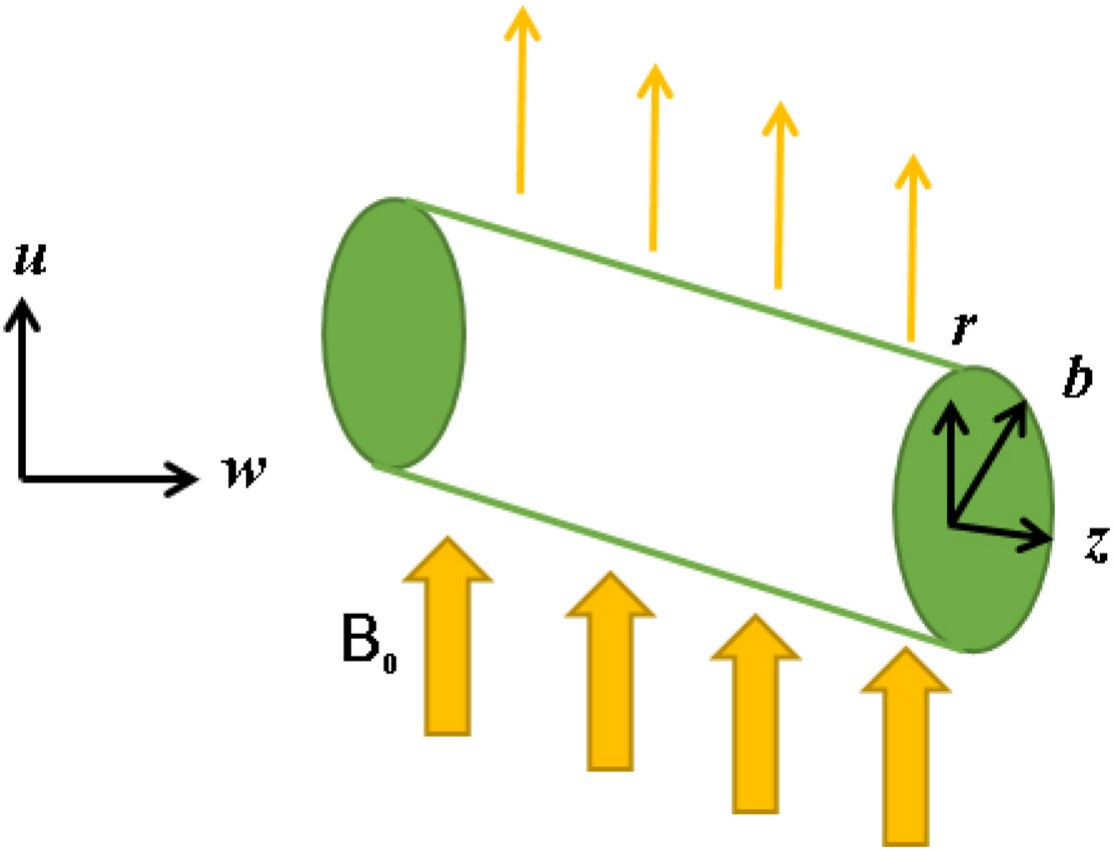
Flow geometry.

An incompressible nanofluid flow comprising carbon nanotubes and ethylene glycol as base fluid past a stretching cylinder with multiple slips is considered here. The coordinates of the cylinder are selected in a way that *r* and* z* in horizontal and vertical directions respectively with cylinder radius *a*. The influence of HOM-HET reactions and C–C heat flux, with magnetic field in a Darcy–Forchheimer permeable medium is also considered. The HOM reaction happens in the liquid while the HET reaction arises on the surface. The layout of the envisioned mathematical model is given in Fig. 1. Chaudhary and Merkin^56^ established the isothermal homogenous reaction with cubic autocatalysis in the nanofluid flow and is described by:

1 $$C + 2D \to 3D,$$

and the heterogeneous reaction is given by:

2 $$C \to D.$$

The rate of homogenous reaction is $k_{1} Hb^{2}$ and rate of heterogeneous reaction is $k_{s} H$. Here, $H$ and $b$ are the chemical species of concentrations $C$ and $D$ respectively.

The governing boundary layer equations depicting the assumed model are^43,57^:

3 $$(rw)_{z} + (ru)_{r} = 0,$$

4 $$\rho_{NF} (ww_{z} + uw_{r} ) = \mu_{NF} \left( {w_{rr} + \frac{1}{r}w_{r} } \right) - \sigma_{F} B_{0}^{2} w - \frac{{\mu_{NF} }}{{k^{*} }}w - Fw^{2} ,$$

5 $$\rho_{NF} (wu_{z} + uu_{r} ) = - P_{r} + \mu_{NF} \left( {u_{rr} + \frac{1}{r}u_{r} - \frac{u}{{r^{2} }}} \right),$$

6 $$\begin{gathered} (wT_{z} + uT_{r} ) = \frac{{k_{NF} }}{{(\rho c_{p} )_{NF} }}\left( {T_{rr} + \frac{1}{r}T_{r} } \right) - \lambda_{2} \left( \begin{gathered} w^{2} T_{zz} + u^{2} T_{rr} + 2uwT_{rz} + ww_{z} T_{z} \hfill \\ + wu_{z} T_{r} + uw_{r} T_{z} + uu_{r} T_{z} + uu_{r} T_{r} \hfill \\ \end{gathered} \right) - \frac{q*}{{(\rho c_{p} )_{NF} }}, \hfill \\ \hfill \\ \end{gathered}$$

7 $$uH_{r} + wH_{z} = D_{A} \left( {H_{rr} + \frac{1}{r}H_{r} } \right) - k_{1} Hb^{2} ,$$

8 $$ub_{r} + wb_{z} = D_{B} \left( {b_{rr} + \frac{1}{r}b_{r} } \right) + k_{1} Hb^{2} ,$$

with the associated suitable conditions:

$$u = 0,\,\,\,\,\,w = W_{w} + Lw_{r} ,\,\,\,\,T = T_{w} + IT_{r} \,\,\,\,D_{A} H_{r} = k_{s} H\,\,\,\,\,D_{B} b_{r} = - k_{s} H\,\text{at}\,r = a$$

9 $$u \to 0,\,\,\,T \to T_{\infty } ,\,\,\,\,H \to a_{0} ,\,\,\,\,\,b \to 0\,\text{as}\,r \to \infty .$$

Tables 2 and 3 represent the thermophysical properties of CNTs and Ethylene glycol.

**Table 2:** The thermophysical characteristics of CNTs are given.

Properties	SWCNT’s and MWCNT’s
Dynamic viscosity	$$A_{1} =$$$$\frac{{\mu_{NF} }}{{\mu_{F} }} = \frac{1}{{(1 - \varphi )^{2.5} }}$$
Heat capacity	$A_{4} = \frac{{(\rho c)_{NF} }}{{(\rho c)_{F} }} = (1 - \varphi ) + \varphi \frac{{(\rho c)_{CNT} }}{{(\rho c)_{F} }}$
Density	$A_{2} = \frac{{\rho_{NF} }}{{\rho_{F} }} = (1 - \varphi ) + \varphi \frac{{\rho_{CNT} }}{{\rho_{F} }}$
Thermal conductivity	$$\begin{gathered} A_{3} = \frac{{k_{NF} }}{{k_{F} }},\frac{{k_{NF} }}{{k_{bF} }} = \left( {\frac{{1 - \varphi_{2} + 2\varphi_{2} \frac{{k_{MWCNT} }}{{k_{MWCNT} - k_{F} }}\ln \left( {\frac{{k_{MWCNT} + k_{F} }}{{2k_{F} }}} \right)}}{{1 - \varphi_{2} + 2\varphi_{2} \frac{{k_{f} }}{{k_{MWCNT} - k_{F} }}\ln \left( {\frac{{k_{MWCNT} + k_{F} }}{{2k_{F} }}} \right)}}} \right) \hfill \\ \frac{{k_{bF} }}{{k_{f} }} = \left( {\frac{{1 - \varphi_{1} + 2\varphi_{1} \frac{{k_{SWCNT} }}{{k_{SWCNT} - k_{F} }}\ln \left( {\frac{{k_{SWCNT} + k_{F} }}{{2k_{F} }}} \right)}}{{1 - \varphi_{1} + 2\varphi_{1} \frac{{k_{f} }}{{k_{SWCNT} - k_{F} }}\ln \left( {\frac{{k_{SWCNT} + k_{F} }}{{2k_{F} }}} \right)}}} \right) \hfill \\ \end{gathered}$$
**For hybrid nanofluid**	
Density	$\rho_{HNF} = \varphi_{2} \rho_{{s_{2} }} + \left\{ {\varphi_{1} } \right.\rho_{{s_{1} }} + (1 - \varphi_{1} )\rho_{F} \left. {(1 - \varphi_{2} )} \right\},$
Heat capacity	$$\begin{gathered} (\rho c_{p} )_{HNF} = \varphi_{2} (\rho c_{p} )_{{s_{2} }} + \left\{ {\varphi_{1} } \right.(\rho c_{p} )_{{s_{1} }} + [(1 - \varphi_{2} )(\rho c_{p} )_{F} \left. {(1 - \varphi_{1} )]} \right\} \hfill \\ \hfill \\ \end{gathered}$$
Variable viscosity	$\mu_{HNF} = \frac{{\mu_{F} }}{{(1 - \varphi_{1} )^{2.5} (1 - \varphi_{2} )^{2.5} }},\,$
Thermal conductivity	$$\begin{gathered} \frac{{k_{HNF} }}{{k_{bF} }} = \frac{{(n - 1)k_{bF} + k_{s_2} - (k_{bF} - k_{s_2} )(n - 1)\varphi_{2} }}{{(n - 1)k_{bF} + k_{s_2} + (k_{bF} - k_{s_2} )\varphi_{2} }} \hfill \\ \frac{{k_{bF} }}{{k_{F} }} = \frac{{(n - 1)k_{F} + k_{s_1} - (k_{F} - k_{s_1} )(n - 1)\varphi_{1} }}{{(n - 1)k_{F} + k_{s_1} + (k_{F} - k_{s_1} )\varphi_{1} }},\,\,\, \hfill \\ \end{gathered}$$

**Table 3:** Thermophysical properties of EG and CNTs^47,50^.

Physical properties	Ethylene glycol	SWCNT	MWCNT
$\rho \left( {\text{kg}\,\text{m}^{ - 3} } \right)$	1115	2600	1600
$c_{p} \left( {\frac{{\text{kg}\,\text{K}}}{\text{J}}} \right)^{ - 1}$	2430	425	796
$K\left( {\frac{{\text{mK}}}{\text{W}}} \right)^{ - 1}$	0.253	6600	3000

By considering the following transformation:

10 $$u = - \frac{ca}{{\sqrt \eta }}F(\eta ),\,w = 2zcF^{\prime}(\eta ),\,\theta (\eta ) = \frac{{T - T_{\infty } }}{{T_{w} - T_{\infty } }},\,\,\Delta T = T_{w} - T_{\infty } \,,\,\eta = \left( \frac{r}{a} \right)^{2} ,g = \frac{H}{{a_{0} }},\,\,\,\,\,h = \frac{b}{{a_{0} }}.$$

Equations (4)–(9) become:

11 $$A_{1} (\eta F^{\prime\prime\prime} + F^{\prime\prime}) + \frac{{A_{2} {\text{Re}} }}{2}(FF^{\prime\prime} - F^{{\prime}{2}} ) - {\text{Re}} F_{r} F^{{\prime}{2}} - \frac{\lambda }{4}\frac{{{\text{Re}} A_{1} }}{{A_{4} }}F^{\prime} - \frac{M}{4}F^{\prime} = 0,$$

12 $$A_{3} (\theta ^{\prime} + 2\eta \theta ^{\prime\prime}) + A*F^{\prime} + B*\theta - 2\gamma Re\Pr \left( {\frac{{F^{2} }}{\eta }\theta ^{\prime\prime} + FF^{\prime}\theta ^{\prime}} \right) - {\text{Re}} A_{4} \Pr F\theta ^{\prime} = 0,$$

13 $$2\eta g^{\prime\prime} + 2g^{\prime} + 2Sc{\text{Re}} Fg^{\prime} - Sc{\text{Re}} Kgh^{2} = 0,$$

14 $$2\eta h^{\prime\prime} + 2h^{\prime} + 2\frac{Sc}{\delta }{\text{Re}} Fg^{\prime} + \frac{Sc}{\delta }{\text{Re}} Kgh^{2} = 0,$$

15 $$\begin{aligned} & F^{\prime}(1) = 1 + SF^{\prime\prime},\,\,\,\,F(1) = 0,\,\,\,\,\,\,\theta (1) = 1 + t\theta ^{\prime}(1)\,\,\,g^{\prime}(1) = - K_{s} g(1),\, \\ & \delta h^{\prime}(1) = - K_{s} g(1)\,,\,F^{\prime}(\infty ) = 0,\,\,\,\,\,\,\,\theta (\infty ) \to 0,\,\,\,\,\,\,g(\infty ) \to 1,\,\,\,\,h(\infty ) \to 0. \\ \end{aligned}$$

The quantities defined above are given as:

16 $$\begin{aligned} & \Pr = \frac{{\upsilon_{F} }}{{\alpha_{F} }},\,\,{\text{Re}} = \frac{{ca^{2} }}{{2\upsilon_{F} }},\lambda = \frac{{a^{2} }}{4k},\delta = \frac{{D_{B} }}{{D_{A} }},\,\,Sc = \frac{\upsilon }{{D_{A} }},\,\,\,K_{s} = \frac{{k_{s} a_{0} }}{{D_{B} }},\,\,\,K = \frac{{k_{1} a_{0}^{2} }}{c},\,\, \\ & S = \frac{2L}{a},t = \frac{2I}{a},\gamma = \lambda_{2} a. \\ \\ \end{aligned}$$

The diffusion Coefficients $D_{A}$ and $D_{B}$ are understood to be the same if $\delta = 1,$ resulting in the subsequent relation:

17 $$g(\eta ) + h(\eta ) = 1.$$

Thus Eqs. (13) and (14) become:

18 $$2\eta g^{\prime\prime} + 2g^{\prime} + 2Sc{\text{Re}} Fg^{\prime} - Sc{\text{Re}} Kg(1 - g)^{2} = 0,$$

with the corresponding boundary conditions:

19 $$g^{\prime}(1) = - K_{s} g(1),\,\,\,\,\,\,\,\,g(\infty ) \to 1.$$

The surface Drag force coefficient is characterized by:

20 $$C_{F} = \frac{{\tau_{w} }}{{\frac{1}{2}\rho_{f} W_{w}^{2} }},$$

where

21 $$\tau_{w} = \mu_{F} \left. {w_{r} } \right|_{r = a} .$$

The dimensionless Skin friction coefficient is appended as under:

22 $$C_{F} = F^{\prime\prime}(1),$$

### Numerical solution

The numerical approach of MATLAB software bvp4c is applied to assess the transmuted ordinary differential equations system. A step size of $h =$ 0.01 is used in the RK-4 method for better approximations. The procedure is valid if the auxiliary terminal conditions are met with an accuracy of $10^{ - 6}$. Initially, additional variables are instituted as:

23 $$\begin{aligned} & F = y_{1} F^{\prime} = y_{2} ,F^{\prime\prime} = y_{3} ,F^{\prime\prime\prime} = y^{\prime}_{3} , \\ & \theta = y_{4} ,\theta^{\prime} = y_{5} ,\theta^{\prime\prime} = y^{\prime}_{5} , \\ & \varphi = y_{6} ,\varphi^{\prime} = y_{7} ,\varphi^{\prime\prime} = y^{\prime}_{7} . \\ \end{aligned}$$

Using Eq. (23), we have

24 $$yy_{1} = (1/\eta A_{1} )\left[ { - A_{1} y_{3} - A_{2} {\text{Re}} (y_{1} y_{3} - y_{2}^{2} ) + {\text{Re}} F_{r} y_{2}^{2} + \frac{{\lambda {\text{Re}} }}{{2A_{4} }}A_{1} y_{2} + My_{2} } \right],$$

25 $$yy_{2} = \left( {\frac{1}{{2\eta A_{3} - 2\gamma {\text{Re}} A_{4} \Pr \frac{{y_{1}^{2} }}{\eta }}}} \right)\left[ \begin{gathered} - A_{3} y_{5} - A*y_{2} - B*y_{4} + 2\gamma {\text{Re}} A_{4} \Pr y_{1} y_{2} y_{5} \hfill \\ + {\text{Re}} A_{4} \Pr y_{1} y_{5} \hfill \\ \end{gathered} \right],$$

26 $$yy_{3} = (1/2\eta )\left[ { - 2y_{7} - 2Sc{\text{Re}} y_{1} y_{7} + Sc{\text{Re}} Ky_{6} (1 - y_{6} )^{2} } \right],$$

with the transformed boundary conditions

$$y_{2} (1) - 1 - Sy_{3} (1);\,\,\,\,\,y_{1} (1);\,\,\,\,\,y_{4} (1) - 1 - ty_{5} (1);\,\,\,\,\,y_{7} (1) - \frac{{K_{s} }}{2\sqrt \eta }y_{6} (1);\,\,$$

27 $$y_{2} (\infty );\,\,\,\,y_{4} (\infty );\,\,\,y_{6} (\infty ) - 1;$$

### Entropy generation analysis

The volumetric entropy generation (*S*_*G*_) is present at^58–63^

28 $$\begin{aligned} S_{G}^{{{\prime \prime \prime }}} & = \underbrace {{\frac{{\kappa_{NF} }}{{T_{\infty }^{2} }}T_{r}^{2} }}_{Heat\,\,transfer\,\,\,\,\,\,\,\,Irreversibility} + \underbrace {{\frac{{\mu_{NF} }}{{T_{\infty } }}w_{r}^{2} }}_{Viscous\,\,\,dissipation\,\,\,\,irreversibility} - \underbrace {{\frac{{\mu_{NF} }}{{T_{\infty } k^{*} }}w}}_{porous \, medium \, irreversibility} + \underbrace {{\frac{{\sigma B_{0}^{2} }}{{T_{\infty } }}w^{2} }}_{{\begin{array}{*{20}l} {Joule \, heating \, irreversibility} \hfill \\ \end{array} }} \\ & \quad + \underbrace {{\frac{{RD_{A} }}{{T_{\infty } }}\left( {H_{r} T_{r} } \right) + \frac{{RD_{A} }}{{a_{0} }}\left( {H_{r} } \right)^{2} + \frac{{RD_{B} }}{{T_{\infty } }}\left( {b_{r} T_{r} } \right) + \frac{{RD_{B} }}{{a_{0} }}\left( {b_{r} } \right)^{2} }}_{mass \, transfer \, irreversibility} \\ \end{aligned}$$

The entropy generation rate is equal to $N_{S} = \frac{{S_{G}^{\prime\prime\prime} }}{{S_{0}^{\prime\prime\prime} }}$

29 $$S_{0}^{\prime\prime\prime} = \frac{{4k_{f} (T_{w} - T_{\infty } )^{2} }}{{L^{2} T_{\infty }^{2} }}.$$

The Entropy generation in the dimensionless form can be derived by using the above transformation which is given in Eq. (10).

30 $$N_{S} = \frac{{S_{G}^{\prime\prime\prime} }}{{S_{0}^{\prime\prime\prime} }} = \left( \begin{gathered} A_{3} \frac{\eta }{m}\theta ^{{\prime}{2}} (\eta ) - A_{1} \frac{\lambda }{4}F^{\prime} + \frac{{A_{1} }}{m}\frac{Br}{\Omega }\eta f^{{\prime\prime}{2}} + \frac{Br}{{m\Omega }}M^{2} f^{^{\prime}2} (\eta ) \hfill \\ + L_{1} \theta ^{\prime}g^{\prime} + \frac{{L_{1} g^{{\prime}{2}} }}{\Omega } + L_{2} \theta ^{\prime}h^{\prime} + \frac{{L_{2} h^{{\prime}{2}} }}{\Omega } \hfill \\ \end{gathered} \right).$$

The diffusion Coefficients $D_{A}$ and $D_{B}$ are understood to be the same if $\delta = 1,$ resulting in the subsequent relation: $g(\eta ) + h(\eta ) = 1.$ Thus Eq. (30) become:

31 $$N_{S} = \frac{{S_{G}^{\prime\prime\prime} }}{{S_{0}^{\prime\prime\prime} }} = \left( \begin{gathered} A_{3} \frac{\eta }{m}\theta ^{{\prime}{2}} (\eta ) - A_{1} \frac{\lambda }{4}F^{\prime} + \frac{{A_{1} }}{m}\frac{Br}{\Omega }\eta f^{{\prime\prime}{2}} + \frac{Br}{{m\Omega }}M^{2} f^{^{\prime}2} (\eta ) \hfill \\ + L_{1} \theta ^{\prime}g^{\prime} + \frac{{L_{1} g^{{\prime}{2}} }}{\Omega } + L_{2} \theta ^{\prime}g^{\prime} + \frac{{L_{2} g^{{\prime}{2}} }}{\Omega } \hfill \\ \end{gathered} \right),$$

32 $$\begin{gathered} m = \frac{{a^{2} }}{{L^{2} }},\,\,\,Br = \frac{{\mu_{f} W_{w}^{2} }}{{k_{F} (T_{w} - T_{\infty } )}},\,\,\,\,\Omega = \frac{{T_{w} - T_{\infty } }}{{T_{\infty } }}\,\,\,L_{1} = \frac{{RD_{A} a_{0} }}{k},\,\,\,\,L_{2} = \frac{{RD_{B} a_{0} }}{k}. \hfill \\ \hfill \\ \end{gathered}$$

To get a similarity solution, all parameters must be constant. The Brinkman number is given by

33 $$Br = \frac{{\mu_{f} W_{w}^{2} (z)}}{{k_{f} \left( {T_{w} - T_{\infty } } \right)}} = \frac{{\mu z^{2} c^{2} }}{{k_{f} \left( {T_{w} - T_{\infty } } \right)}}.$$

For *Br* to be independent of *z,* the temperature $T_{w} (z)$ should be in the form $T_{w} = T_{\infty } + T_{o} z^{2}$. Where $T_{o}$ is constant, otherwise the solution obtained is only locally similar. By using the above define transformation the Eq. (31) becomes:

34 $$Br = \frac{{\mu c^{2} }}{{k_{f} \left( {T_{w} - T_{\infty } } \right)}}.$$

The Bejan number (*Be*) is presented as:

*Be* = $$\frac{\mathrm{Heat and mass transfer irreversibility}}{\mathrm{total entropy}}$$

35 $$Be = \frac{{\underbrace {{\frac{{\kappa_{NF} }}{{T_{\infty }^{2} }}T_{r}^{2} }}_{Heat\,\,transfer\,\,\,\,\,\,\,\,Irreversibility} + \underbrace {{\frac{{RD_{A} }}{{T_{\infty } }}\left( {H_{r} T_{r} } \right) + \frac{{RD_{A} }}{{a_{0} }}\left( {H_{r} } \right)^{2} + \frac{{RD_{B} }}{{T_{\infty } }}\left( {b_{r} T_{r} } \right) + \frac{{RD_{B} }}{{a_{0} }}\left( {b_{r} } \right)^{2} }}_{mass \, transfer \, irreversibility}}}{\begin{gathered} \underbrace {{\frac{{\kappa_{NF} }}{{T_{\infty }^{2} }}T_{r}^{2} }}_{Heat\,\,transfer\,\,\,\,\,\,\,\,Irreversibility} + \underbrace {{\frac{{\mu_{NF} }}{{T_{\infty } }}w_{r}^{2} }}_{Viscous\,\,\,dissipation\,\,\,\,irreversibility} - \underbrace {{\frac{{\mu_{NF} }}{{T_{\infty } k^{*} }}w}}_{porous \, medium \, irreversibility} + \underbrace {{\frac{{\sigma B_{0}^{2} }}{{T_{\infty } }}w^{2} }}_{{\begin{array}{*{20}l} {Joule \, heating \, irreversibility} \hfill \\ \end{array} }} \hfill \\ + \underbrace {{\frac{{RD_{A} }}{{T_{\infty } }}\left( {H_{r} T_{r} } \right) + \frac{{RD_{A} }}{{a_{0} }}\left( {H_{r} } \right)^{2} + \frac{{RD_{B} }}{{T_{\infty } }}\left( {b_{r} T_{r} } \right) + \frac{{RD_{B} }}{{a_{0} }}\left( {b_{r} } \right)^{2} }}_{mass \, transfer \, irreversibility}. \hfill \\ \end{gathered} }$$

In the dimensionless form, the *Be* is presented as:

36 $$\begin{gathered} Be = \frac{{A_{3} \frac{\eta }{m}\theta ^{{\prime}{2}} (\eta ) + L_{1} \theta ^{\prime}g^{\prime} + \frac{{L_{1} g^{{\prime}{2}} }}{\Omega } + L_{2} \theta ^{\prime}g^{\prime} + \frac{{L_{2} g^{{\prime}{2}} }}{\Omega }}}{\begin{gathered} A_{3} \frac{\eta }{m}\theta ^{{\prime}{2}} (\eta ) + \frac{{A_{1} }}{m}\frac{Br}{\Omega }\eta f^{{\prime\prime}{2}} - A_{1} \frac{\lambda }{4}F^{\prime} + \frac{Br}{{m\Omega }}M^{2} f^{{\prime}{2}} (\eta ) \hfill \\ + L_{1} \theta ^{\prime}g^{\prime} + \frac{{L_{1} g^{{\prime}{2}} }}{\Omega } + L_{2} \theta ^{\prime}g^{\prime} + \frac{{L_{2} g^{{\prime}{2}} }}{\Omega }. \hfill \\ \end{gathered} } \hfill \\ \hfill \\ \end{gathered}$$

## Results and discussion

**Figure 2:**
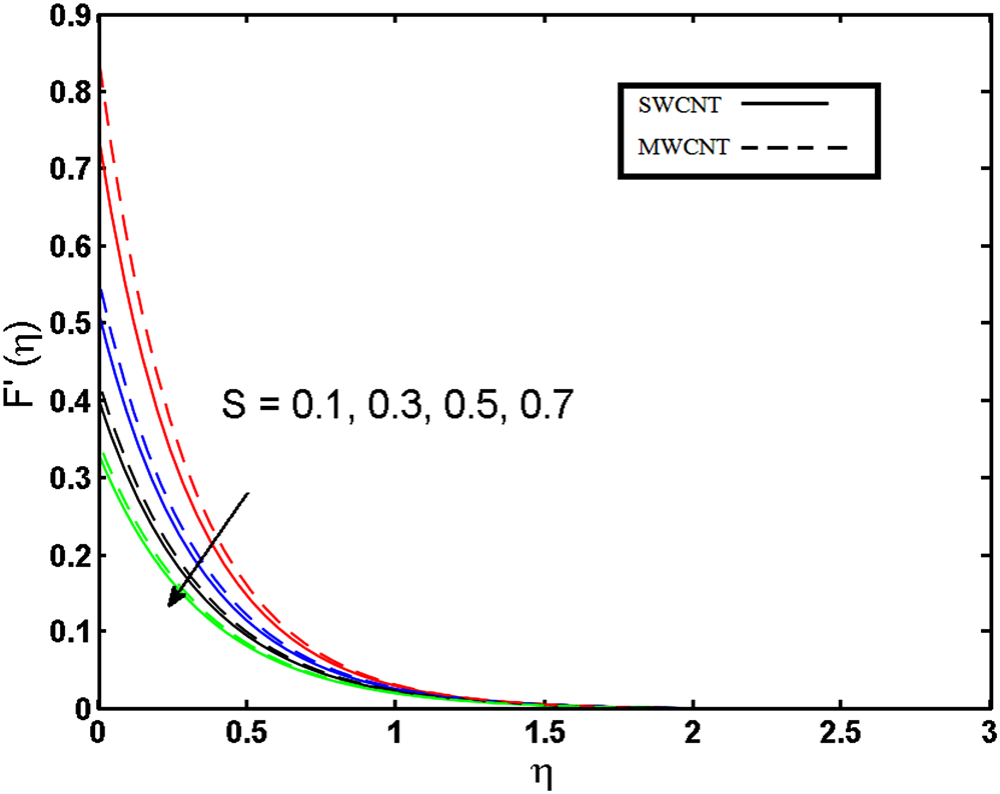
Illustration of a velocity profile $F^{\prime}(\eta )$ for different values of $S$.

**Figure 3:**
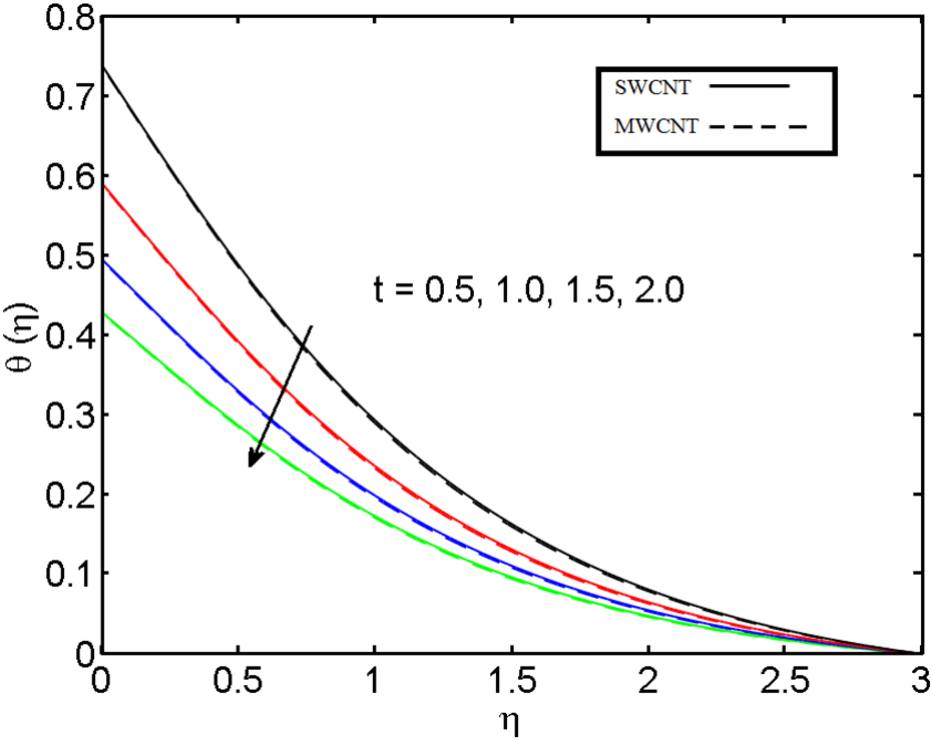
Illustration of thermal profile $\theta (\eta )$ for different values of $t$.

**Figure 4:**
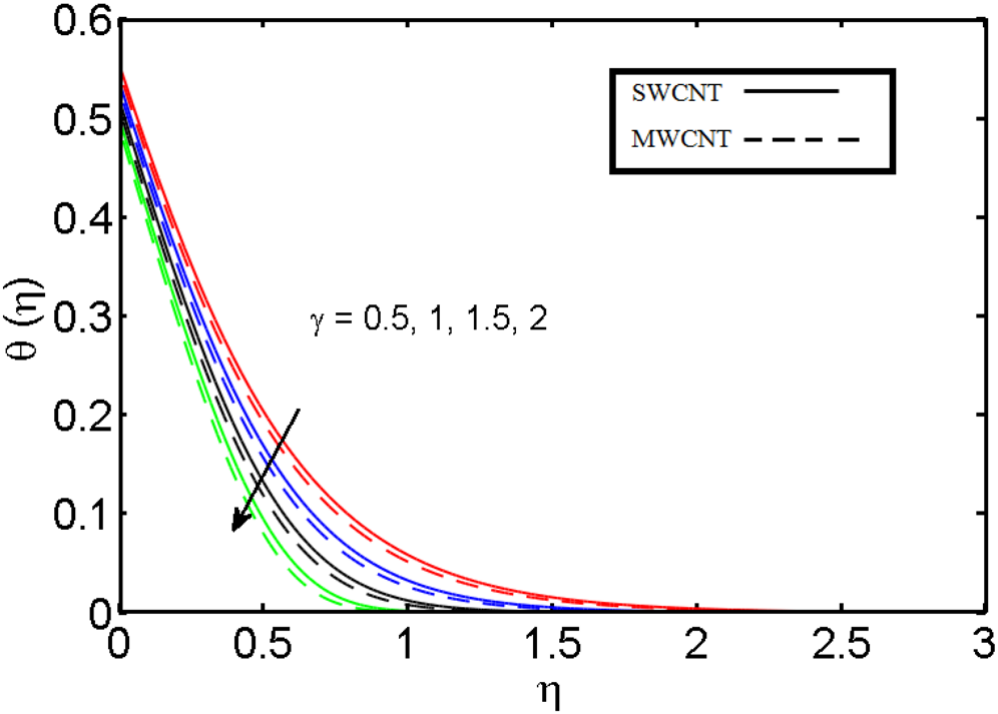
Illustration of thermal profile $\theta (\eta )$ for different values of $\gamma$.

**Figure 5:**
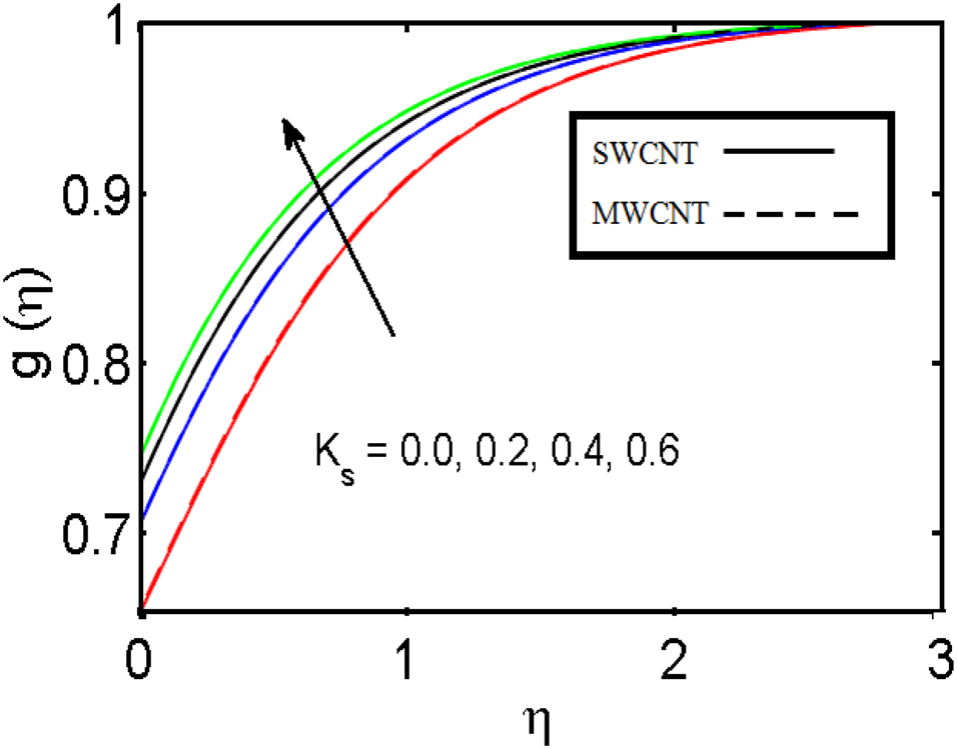
Illustration of concentration profile $g(\eta )$ for different values of $K_{s}$.

**Figure 6:**
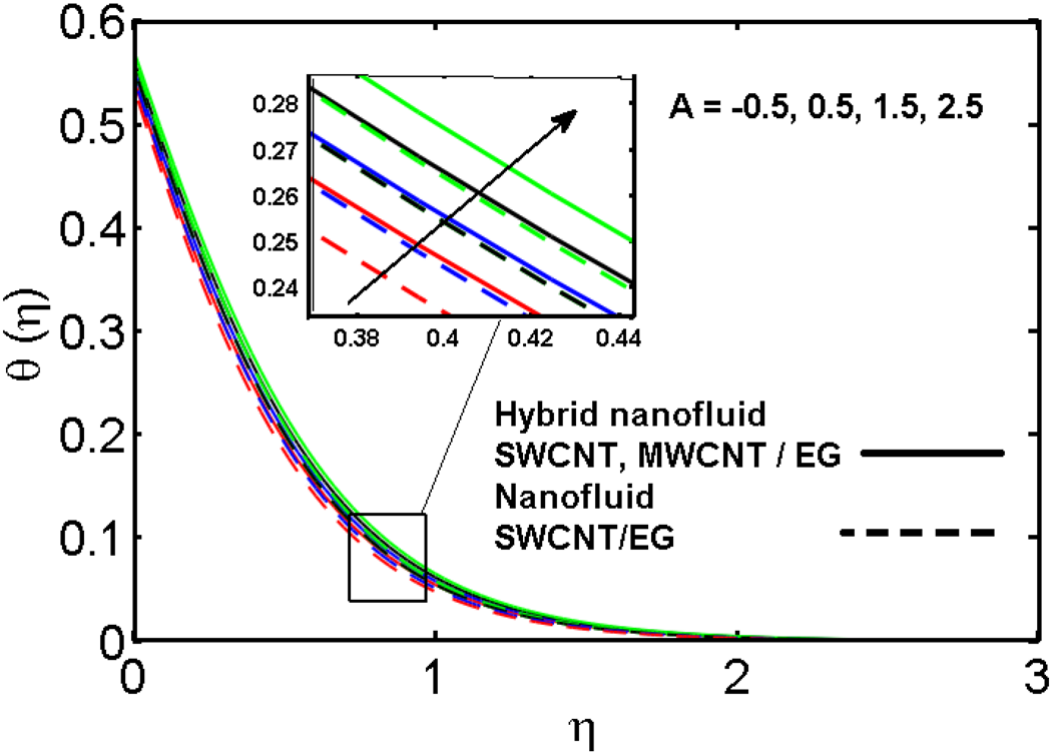
Illustration of concentration profile $\theta (\eta )$ for different values of $A$.

**Figure 7:**
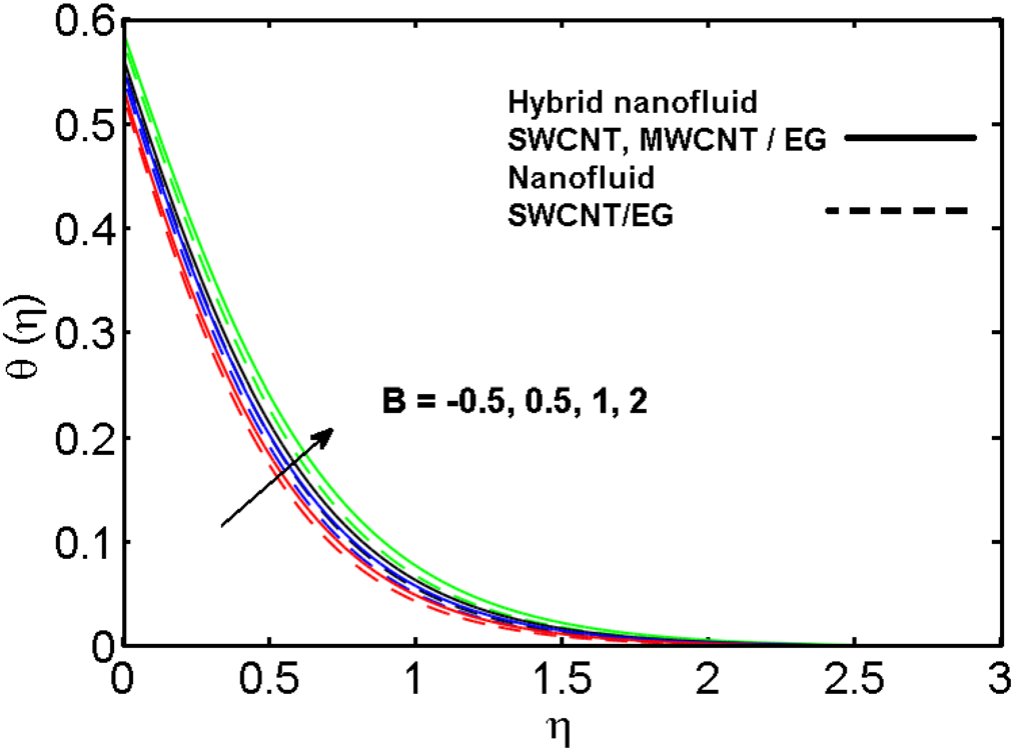
Illustration of concentration profile $\theta (\eta )$ for different values of $B$.

**Figure 8:**
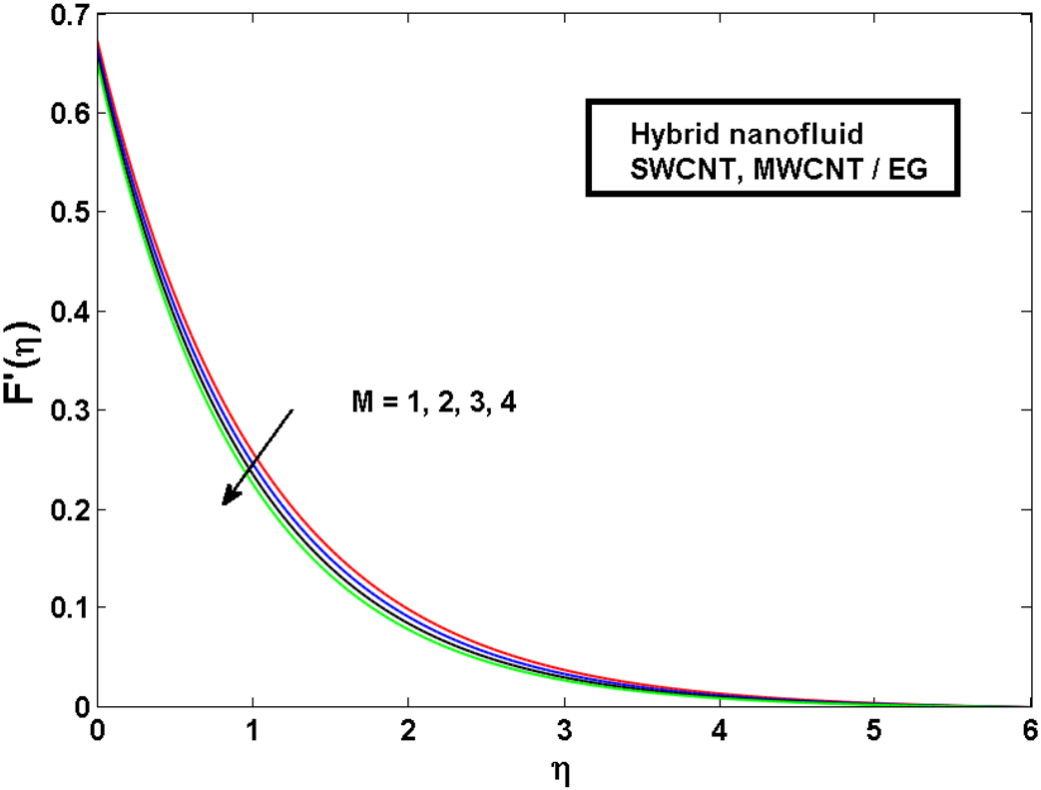
Illustration of a velocity profile $F^{\prime}(\eta )$ for different values of $M$.

**Figure 9:**
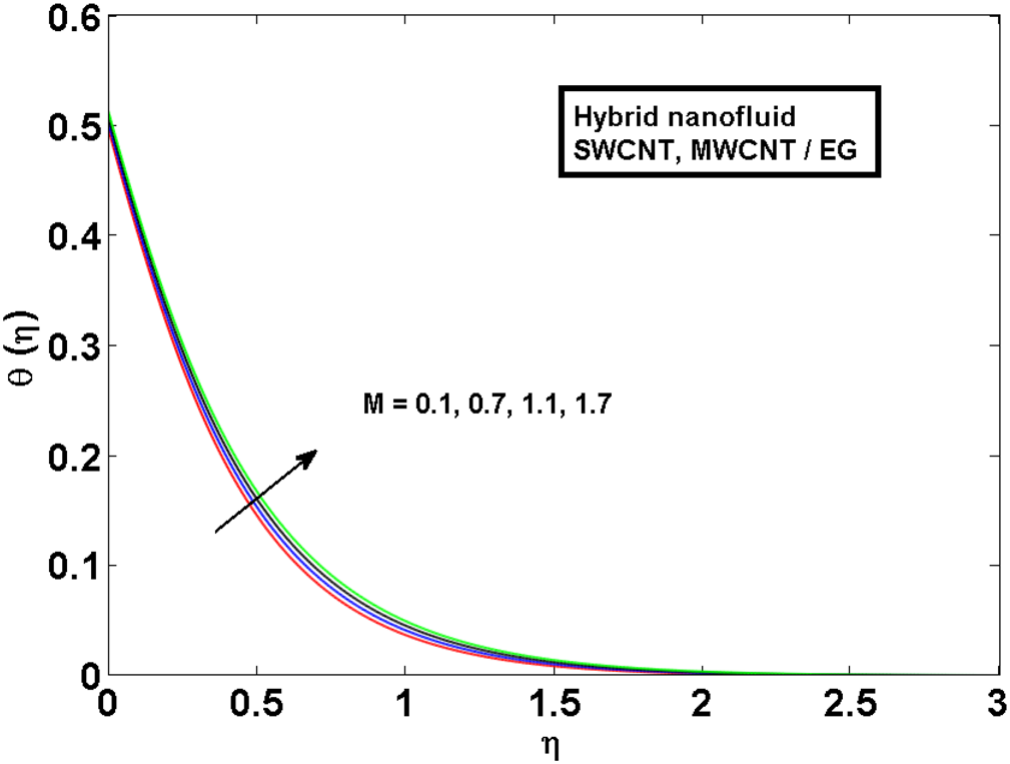
Illustration of thermal profile $\theta (\eta )$ for different values of $M$.

**Figure 10:**
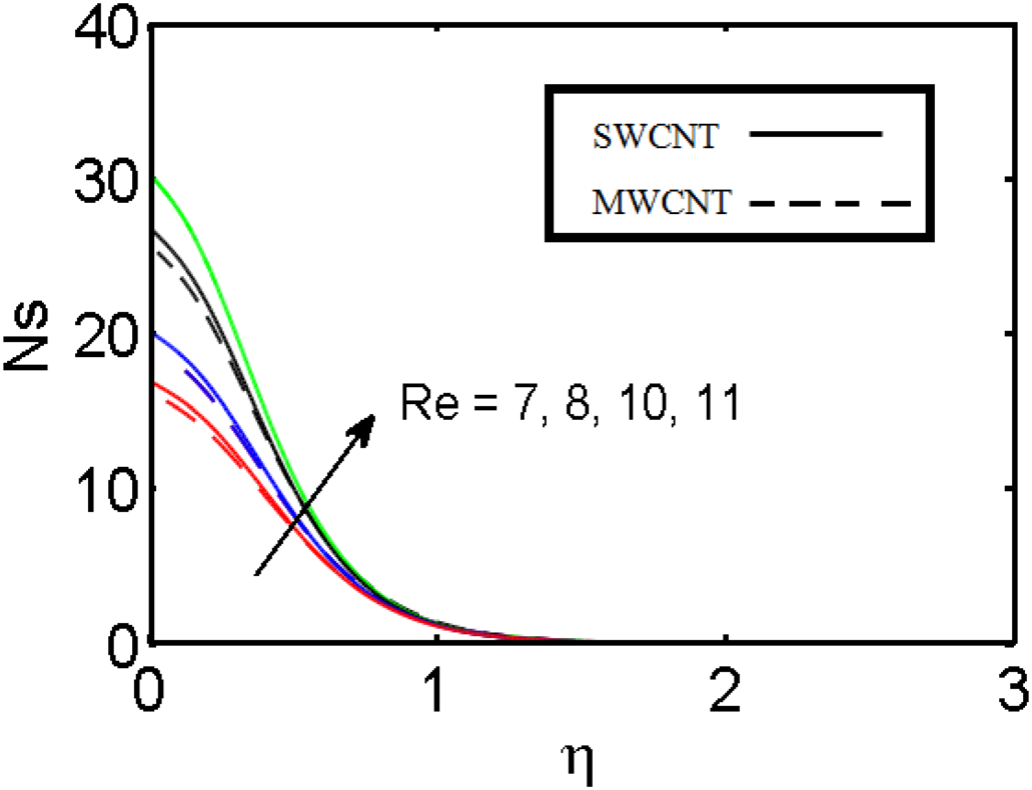
Illustration of entropy generation $Ns$ for different values of ${\text{Re}}$.

**Figure 11:**
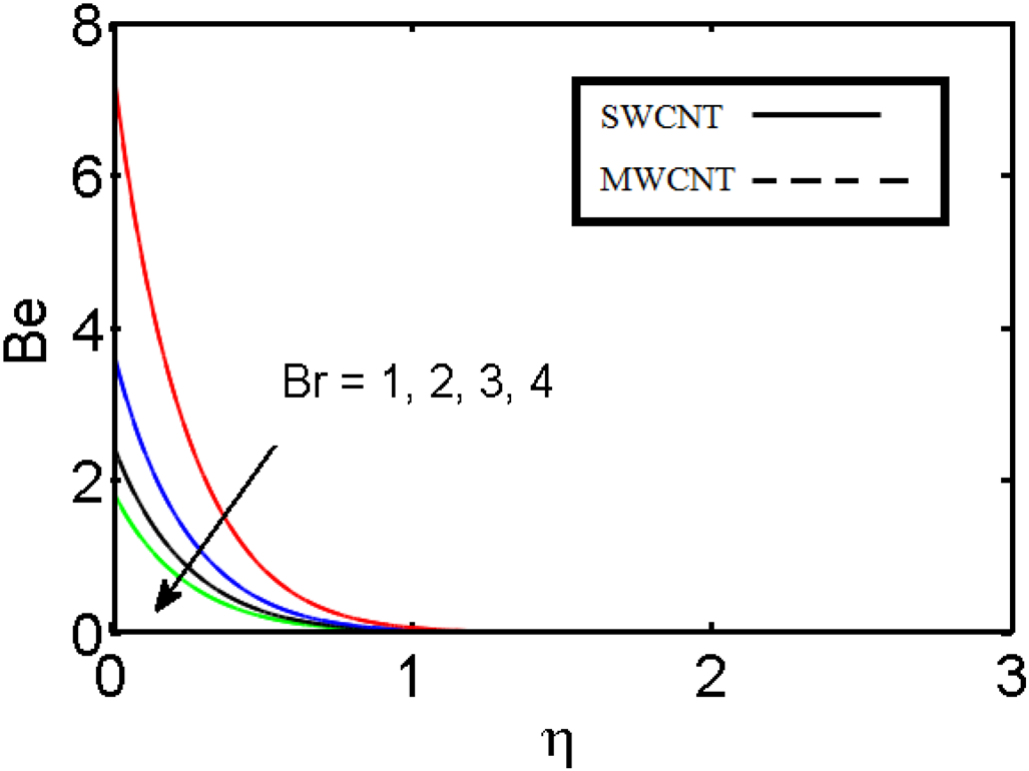
Illustration of Bejan number $Be$ for different values of $Br$.

This section (Figs. 2, 3, 4, 5, 6, 7, 8, 9) is devised to examine the noticeable features of the prominent evolving parameters on the associated profiles. Figure 2 depicts the effect of the velocity slip parameter $S$ on the velocity profile. The boundary layer thickness and velocity are found to decrease as the velocity slip parameter is increased. When the velocity slip parameter increases, some of the stretching velocity is shifted to the fluid. Consequently, the velocity profile reduces. Figure 3 shows the effect of the thermal slip parameter (*t*) on the temperature profile. The temperature and thickness of the thermal boundary layer were seen to decrease as the thermal slip parameter is increased. Heat transfer from the cylinder to the neighboring fluid reduces as the thermal slip parameter increases. Consequently, the fluid's temperature drops. The influence of the thermal convection parameter $\gamma$ on the temperature field is demonstrated in Fig. 4. It is noted that both SWCNTs and MWCNTs and thickness of the thermal boundary layer are diminished for growing estimates of $\gamma$. Large values of relaxation time result in non-conductive behavior of the material which is liable for a decline in the temperature field. Figure 5 address the impact of the HET reactions ${K}_{s}$ on concentration field. Here one can observe that the concentration field increases with greater estimations of *K*_*s*_ for both multi and single-wall CNTs. This impact happens only in the vicinity of the wall. It is also observed that the MWCNT’s gain a higher heat transfer rate as compared to SWCNTs. Figures 6 and 7 are illustrated to analyze the impact of non-uniform source/sink terms $A$ and $B$ for temperature distribution. For higher values of ($A,B > 0$), a substantial increase in temperature field is detected for both parameters $A$ and $B$. Since the presence of heat source parameter produces more heat within the nanofluid flow field which leads to an increment in the boundary layer thickness. Hence, more heat in the nanofluid enhances the fluid temperature and the same result can be perceived in the case of $B$(Fig. 10). Here, in Figs. 6 and 7, it is observed that the influence of the Hybrid nanofluid is leading as compared to the SWCNTs. The impact of magnetic field on the velocity and temperature profiles for hybrid nanofluid (MWCNT, SWCNT/EG) is shown in Figs. 8 and 9 respectively. Hybrid nano liquid is used to improve the efficiency of cooling processes in the industry. It is observed in Figs. 8 and 9 that hybrid nanoliquid is a combination of nanoparticles (SWCNTs and MWCNT’s) and base fluid (EG). The velocity profile is considered to indicate decreasing curves for different estimations of *M*. Physically for strong Lorentz force causes resistance in a fluid motion and the fluid becomes less viscous that’s why the velocity profile reduces. The impact of the magnetic field parameter on the temperature profile is given in Fig. 9. As the magnetic field parameter rises, the thickness of the thermal boundary layer rises, which causes an increase in the thermal profile. Figures 10 and 11 display the role of volumetric entropy generation (*S*_*G*_) discussed for $Br$ and *Re*. The effect of *Re* on *Ns* is expressed in Fig. 10. For large estimates of the Reynolds number, the substantial motion of the fluid molecules is witnessed. Thus, escalating the entropy generation rate (*S*_*G*_). Figure 11 is drawn to visualize the influence of $Br$ versus Bejan number *Be*. It is seen that *Be* is the diminishing function of the $Br$. For $Br = 0,$ the irreversibility is dependent only on the factor of heat transfer. Because irreversibility of viscous dissipation is ended and the only irreversibility of heat transfer holds.

Table 4 is erected to witness the influence on the surface Drag force coefficient $C_{Fx}$ for large estimates of $M,\lambda ,{\text{Re}} ,F_{r}$. It is observed that the $C_{Fx}$ escalates versus growing values of all parameters $M,\lambda ,{\text{Re}} ,F_{r}$. Table 5 depicts a comparison for varied values of *M* with Ishak et al.^34^ and Gholinia et al.^35^ in limiting case. An excellent association between the outcomes is achieved.

**Table 4:** Numerical results of Drag force $C_{F} {\text{Re}}_{{}}^{1/2}$ versus the different estimations of the mounting estimates of the $M,\lambda ,{\text{Re}} ,F_{r}$.

1	${\text{Re}}$	$F_{r}$	$\lambda$	$- \sqrt {\text{Re}} C_{F}$
0.5	5.0	0.3	2	1.06688
1.0				1.07759
1.5				1.08786
2.0				1.09770
	5.0			1.06688
	6.0			1.10219
	7.0			1.13230
	8.0			1.15849
	3.0	0.3		1.06688
		0.5		1.07322
		0.7		1.07933
		0.9		1.08523
		0.3	1.0	0.99260
			2.0	1.06688
			3.0	1.12375
			4.0	1.16944

**Table 5:** Comparison and error analysis of current work with published works Ishak et al.^64^ and Gholinia et al.^65^ when $S = 0, t = 0, K_{s} = 0,F_{r} = 0$. It is observed from the error analysis (Ishak et al.^64^ and present work) for growing values of magnetic parameter *M* the error is increased, while it is constant for the second case (Gholinia et al.^65^ and present work). It is noticed for the seconds case that the result is approximately equal, and less error is observed here.

$\text{M}$	$- f^{\prime\prime}(1)$					
	^64^	Present result	Error %	^65^	Present result	Error %
0.00	2.4174	2.4021	+ 0.000153%	2.4023	2.4021	+ 0.000002%
0.01	2.4199	2.4023	+ 0.000176%	2.4025	2.4023	+ 0.000002%
0.05	2.4296	2.4100	+ 0.000196%	2.4101	2.4100	+ 0.000001%
0.10	2.4417	2.4196	+ 0.000221%	2.4198	2.4196	+ 0.000002%
0.50	2.5352	2.4986	+ 0.000366%	2.4988	2.4986	+ 0.000002%

## Final remarks

In the current study, we have analyzed the flow of the hybrid nanofluid in Darcy Forchheimer permeable media with multiple slips over a stretching cylinder. The uniqueness of the flow model is enhanced with the addition of modified Fourier law and HOM–HET chemical reaction. The entropy generation analysis of the envisaged mathematical model is also conducted. The numerical results of the dimensionless model are found using MATLAB function bvp4c. The significant observations of the model are added as follows:

- The fluid velocity and temperature are diminishing functions of the velocity and thermal slips parameters respectively.
- The velocity profile is a declining function of magnetic parameter and porosity number. However, an opposing trend is witnessed in the case of fluid temperature.
- The fluid temperature is increased for high estimates of the heat source-sink and nanoparticle volume concentration parameters.
- For higher estimates of Schmidt number and heterogeneous catalyst parameter, strong concentration is observed.

## Nomenclature

$$a_{0} ,a$$: Dimensional constant
$\mu_{NF}$: Viscosity of nanofluid
*Pr*: Prandtl number
$k_{NF}$: Thermal conductivity of nanofluid
$\lambda_{2}$: Thermal relaxation coefficient
$$h_{w}$$: Mass flux
$$T$$: Liquid temperature
$$\varphi$$: Nanoparticle volumetric
$$D_{A}$$*,*$$D_{B}$$: Diffusion coefficients
$\mu_{F}$: Dynamic viscosity of the liquid
$W_{w}$: Stretching linear velocity
$L$: Velocity slip factor
$\gamma$: Thermal relaxation parameter
$M$: Magnetic field parameter
$Ns$: Total entropy generation
$k_{s}$: Coefficient of heterogeneous reaction
$(c_{p} )_{NF}$: Specific heat capacity of nanofluid
*C*_*F*_: Drag force
$(\rho c)_{NF}$: Ratio of heat capacity
*T*_*w*_: Temperature on wall
$$S,t$$: Slip parameters
$$Sh_{x}$$: Sherwood number
$\rho_{NF}$: Density of nanofluid
$$F$$: Base fluid
$(u,w)$: Components of velocities
$$\nu_{F}$$: Kinematic viscosity
$$CNT$$: Carbon nanotubes
$I$: Thermal slip factor
$$k_{F}$$: Thermal conductivity
*Be*: Bejan number
$S_{G}^{{\prime\prime\prime}}$: Entropy generation rate
$L_{1}$: Diffusion rate of homogenous reaction
$K$: Strength of homogenous reaction
$\delta$: Heat absorption parameter
$$F_{r}$$: Inertial coefficient
$K_{s}$: Strength of heterogeneous reaction
$$\tau_{w}$$: Shear stress
$k_{1}^{{}}$: Chemical reaction parameter
$$Sc$$: Schmidt number
$${\text{Re}}$$: Reynold number
$S$: Velocity slip parameter
$$\lambda$$: Porosity parameter
$m$: Dimension less constant
$S_{0}^{\prime\prime\prime}$: Characteristic entropy generation
$\Omega$: Non-dimensionless temperature-different parameter
$$Br$$: Brinkman number
$A*,B*$: Space and temperature-dependent heat generation and absorption coefficients
$L_{2}$: Diffusion rate of homogenous reaction
